# Treating cofactors can reverse the expansion of a primary disease epidemic

**DOI:** 10.1186/1471-2334-10-248

**Published:** 2010-08-23

**Authors:** Lee R Gibson, Bingtuan Li, Susanna K Remold

**Affiliations:** 1 Department of Mathematics, 328 Natural Sciences Bldg., University of Louisville, Louisville, KY 40292 USA; 2 Department of Biology, 139 Life Sciences Bldg., University of Louisville, Louisville, KY 40292 USA

## Abstract

**Background:**

Cofactors, "nuisance" conditions or pathogens that affect the spread of a primary disease, are likely to be the norm rather than the exception in disease dynamics. Here we present a "simplest possible" demographic model that incorporates two distinct effects of cofactors: that on the transmission of the primary disease from an infected host bearing the cofactor, and that on the acquisition of the primary disease by an individual that is not infected with the primary disease but carries the cofactor.

**Methods:**

We constructed and analyzed a four-patch compartment model that accommodates a cofactor. We applied the model to HIV spread in the presence of the causal agent of genital schistosomiasis, *Schistosoma hematobium*, a pathogen commonly co-occurring with HIV in sub-Saharan Africa.

**Results:**

We found that cofactors can have a range of effects on primary disease dynamics, including shifting the primary disease from non-endemic to endemic, increasing the prevalence of the primary disease, and reversing demographic growth when the host population bears only the primary disease to demographic decline. We show that under parameter values based on the biology of the HIV/*S. haematobium *system, reduction of the schistosome-bearing subpopulations (e.g. through periodic use of antihelminths) can slow and even reverse the spread of HIV through the host population.

**Conclusions:**

Typical single-disease models provide estimates of future conditions and guidance for direct intervention efforts relating only to the modeled primary disease. Our results suggest that, in circumstances under which a cofactor affects the disease dynamics, the most effective intervention effort might not be one focused on direct treatment of the primary disease alone. The cofactor model presented here can be used to estimate the impact of the cofactor in a particular disease/cofactor system without requiring the development of a more complicated model which incorporates many other specific aspects of the chosen disease/cofactor pair. Simulation results for the HIV/*S. haematobium *system have profound implications for disease management in developing areas, in that they provide evidence that in some cases treating cofactors may be the most successful and cost-effective way to slow the spread of primary diseases.

## Background

A central goal of modeling disease epidemiology is to predict the long-term fate of pathogens in their host populations. One persistent challenge in this process is to explain marked differences in the progression of the same pathogen through different host populations. For example, models of the beginning of an HIV epidemic must apply to populations where expansion of the pathogen differs dramatically (e.g. in North America where incidence eventually peaked at under 1% per year vs. in sub-Saharan Africa, with a peak incidence 8 times as high [1]). Cofactors, defined as conditions or pathogens that affect the epidemiology of a disease caused by an organism of primary interest, can help to explain such variability in disease progression. Host populations that differ with respect to the rate of spread of a primary disease often differ with respect to ecological factors that indirectly affect disease spread as well.

Cofactors can affect the rate of spread of the primary infection in two ways. First, susceptible hosts affected by a cofactor can experience *enhanced susceptibility *to infection by the primary disease-causing organism, because many cofactors lower the capacity of the host immune system to exclude the pathogen. Second, hosts that bear both the primary infection and the cofactor can have *enhanced infectivity *towards their susceptible neighbors. This occurs when the presence of the cofactor increases the load of circulating primary-pathogen or changes behavior of infected hosts. The effects of enhanced susceptibility and infectivity are incorporated separately in our model.

Cofactors can be behavioral, environmental, genetic or infectious. For example, for HIV, all four types of cofactors have been described. Behavioral cofactors of HIV include risky sexual behavior and IV drug use and can affect both susceptibility and infectivity, e.g. through their direct effects on exposure probabilities. Nutritional status (e.g. degree of caloric, vitamin A, iron, zinc malnutrition) comprises an environmental HIV cofactor, which can increase infectivity among infected hosts when the deficiency increases viral load, and can increase susceptibility by compromising immune function [2,3]. Although the causal mechanisms are not always clear, some kinds of malnutrition are correlated with changes in transmission as well [4,5]. In populations with genetic variability at the CCR5 locus, the wildtype allele (as opposed to the D32 allele) is a genetic cofactor for HIV. It affects acquisition by allowing more entry of HIV into CD4+ T cells, relative to D32 [6]. Finally, a number of pathogens have been identified as cofactors for HIV spread, including herpes simplex virus (HSV)-2 [7,8], hepatitis C virus, human papilloma virus, *Mycobacterium tuberculosis*, malaria causing *Plasmodium*, and *Schistosoma haematobium*, the causal agent of genital schistosomiasis [[9], and refs within].

Cofactors play important roles in a wide variety of other interactive systems as well, including a variety of human and other mammalian hosts (e.g. [10-12]; reviewed in [13]); in insects [14], and in plants [15]. These examples highlight the pervasive nature of cofactor conditions within epidemiological systems. In particular, in natural plant and animal populations, where coinfections may be common [13,15] and in human populations in which access to health care is limited, influences of nutritional and infectious cofactors on disease dynamics may more often be the norm than the exception.

Stratified models involving large numbers of subpopulations (including stratification by sex, age, risk, and disease progression) tailored to particular host populations and their cofactors indicate that the latter can have substantive epidemiological impacts. [7,8,16,17]. Here we analyze a "simplest possible" model built on a classical Susceptible-Infected (SI) framework [18-20] to explore how cofactors can influence the fate of a primary disease and its impact on the host population in the absence of any additional compartmental model structure. This model can be applied to a wide range of systems involving a cofactor and a primary disease. We apply this model to an HIV epidemic in the presence of the cofactor *Schistosoma haematobium *in sub-Saharan Africa, compare our results to a related model without cofactor dynamics, and assess the degree to which treatment of the cofactor can slow the spread of the primary infection.

## Methods

### Transitions among model subpopulations

**Figure 1:**
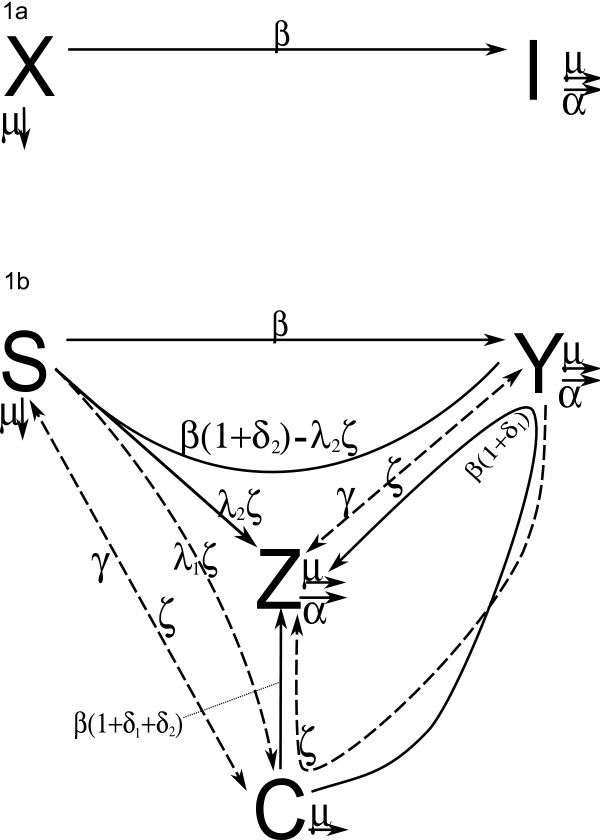
**(a) two-patch SI model and (b) four-patch cofactor model diagrams**. (a) The two-patch SI model appearing in Anderson et. al. (1988) [18], upon which the four-patch cofactor model in (b) is built, and (b) The four-patch cofactor model. Dotted lines represent transitions related to the cofactor condition; solid lines represent transitions related to the primary disease infected population. Curved lines begin and end at the subpopulations from and to which a transition is occurring, and pass near the interacting population which causes the transition.

Our four-patch cofactor model builds upon and inherits all of the parameter names (Table 1) from a two-patch SI model describing changes in susceptible (*X*(*t*)) and infected (*I*(*t*)) subpopulations of a population experiencing an early HIV epidemic ([18], Figure 1a). We therefore begin by describing the parameters which appear in that model. The parameter *α *measures the AIDS related death rate, while deaths from other causes occur at rate *μ*. The per capita birth rate of the population is the constant *ν*, and the net birth rate at time *t *is adjusted to take into account that a fraction (1 - *ϵ*) of offspring of the infected individuals acquire the infection by vertical transmission and die before contributing to the dynamics of the model - effectively at birth. The per capita rate of infection is assumed to be *β *times the infected fraction of the population.

**Table 1:** Parameters appearing in the two-patch SI model and the four-patch cofactor model.

two-patch and four-patch cofactor:	
*μ*	disease-free death rate
*α*	disease-related death rate
*ν*	per capita birth rate
*β*	disease acquisition contact rate
*ϵ*	disease-free fraction of offspring from infected mothers
	
four-patch cofactor only:	

*ζ*	cofactor acquisition contact rate
γ	cofactor recovery rate
*δ*_1_	cofactor induced increase in susceptibility
*δ*_2_	cofactor induced increase in transmission (eg. via increased pathogen concentration)
*λ*_1_	rate adjustment for cofactor acquisition only from SZ contact
*λ*_2_	1 - *λ*_1_

The presence of a cofactor and its biotic interactions with the primary disease organism and with the host results in the need to incorporate new parameters and transitions (Figure 1b). The cofactor spreads according to a contact process with rate *ζ*, and recovery from the cofactor occurs at a constant rate *γ*, e.g. by some form of treatment. We make two simplifying assumptions: that neither acquisition of or recovery from the cofactor condition is affected by infection status with regard to the primary infection. These assumptions yield the simplest possible system that can be used to measure the impact of the cofactor's presence on the primary disease prevalence. Furthermore, because in our model the rate of spread of the cofactor is not exacerbated by the presence of the primary disease, the model reflects a conservative estimate of the impact that the cofactor might make on the progression of the primary disease.

Transitions into and out of subpopulations involving cofactor infections (described below, Results and Discussion) are shown in Figure 1b by the five dotted arrows labeled by either γ or ζ, which appear on the left diagonal (when individuals from *S *and *C *interact), on the arrows between *Y *and *Z *(when individuals from *Y *and *Z *interact), and on the arrow from *Y *which passes near *C *and terminates at *Z *(when individuals from *Y *and *C *interact and the cofactor is transmitted to the individual from *Y *). The interaction between individuals from *S *and *Z *which can result in cofactor transmission to the individual from *S *either with or without the primary infection is explained below.

There are several events by which an individual may contract the primary infection, all of which require contact between an infected individual and an infection-free individual. First, a susceptible individual (from *S*) may interact with a person bearing the primary infection only (from *Y *). The rate at which such individuals contract the primary infection based on this type of contact is described by parameter *β*. This is the only transition inherited from the two-patch model (Figures 1a and 1b top of the triangle (*S *→ *Y *)). Individuals acquiring the primary infection in this manner move from the *S *to the *Y *patch.

Second, a susceptible individual who has the cofactor (from *C*) may interact with a individual with the primary infection only (from *Y *). Because cofactors can increase susceptibility to infection, we incorporate the increase in the rate of transmission due to greater propensity to acquire the primary infection caused by carriage of the cofactor through the parameter *δ*_1_. The overall rate at which a contact between a *C *and a *Y *results in primary disease acquisition by the *C *becomes *β*(1 + *δ*_1_). Cofactor-possessing individuals who contract the primary infection (in this case from contact with an individual from *Y *) must move from subpopulation *C *into the subpopulation *Z *of individuals who have the cofactor and the primary infection. This interaction is represented by the curved arrow which originates at *C *and terminates at *Z *after passing near *Y *(Figure 1b).

Third, an individual who has the cofactor (from *C*) can interact with an individual with both the cofactor and the primary infection (from *Z*). In this case, the rate at which the primary infection is transmitted can be increased both by cofactor-enhanced susceptibility (factor *δ*_1_) and by the cofactor-enhanced infectivity (factor *δ*_2_) resulting in an infection rate of *β*(1 + *δ*_1 _+ *δ *_2_). This also results in a transition from the *C *to the *Z *patch.

Finally, a susceptible individual (from *S*) may interact with a person with both the primary infection and the cofactor (from *Z*). This can result in one of three possible outcomes, depending on whether the cofactor alone (*S *to *C*), the primary infection alone (*S *to *Y *) or both (*S *to *Z*) are transmitted. These three possible transitions are represented by the three central arrows leaving *S *(Figure 1b). Each of these outcomes is described by a different rate in the model. The transmission of the primary disease can be increased (which we describe by a multiplicative factor (1 + *δ*_2_)) by greater propagule production caused by the cofactor, such that the total rate at which individuals from *S *acquire the primary infection and therefore move to either *Y *or *Z *due to interactions with *Z *individuals must be *β*(1 + *δ*_2_). However, susceptible individuals from *S *also contract the cofactor at rate *ζ *from interacting with individuals from *Z*. Thus the total rate at which individuals from *S *move to either *C *or *Z *due to interactions with individuals from *Z *must be *ζ*. We therefore take the rate at which individuals move from *S *to *C*, *Z*, and *Y *due to interaction with individuals from *Z *to be, respectively, λ_1_ζ, λ_2_ζ, and *β*(1 + *δ*_2_) - *λ*_2_*ζ*, where *λ*_1 _+ *λ*_2 _= 1, and 0 ≤ λ_2 _≤ max {1, *β*(1 + *δ*_2_)/ζ}. This choice allows the model to describe either disease/cofactor pairs for which the cofactor and disease may be transmitted together (when *λ*_2 _> 0, for example when both the cofactor and the primary infection are sexually transmitted), or those pairs for which only a single transmission of either the cofactor or the disease may take place during a single encounter (setting *λ*_2 _= 0, e.g. sexually transmitted primary disease, non-sexually transmitted cofactors).

### Cofactor model system equations

The preceding discussion provides the complete rationale for Figure 1b, and therefore also for the following non-linear system of ordinary differential equations. Because the variable *S *= *N *- *Y - Z - C*, no equation is necessary for modeling the behavior of *S*.

(1) $$\begin{array}{l} \frac{dN}{dt}=\nu \;[N-(1-\epsilon )(Y+Z)]-\mu N-\alpha (Y+Z)\\ \frac{dC}{dt}=C\left[-\mu -\gamma -\beta (1+\delta_{1})\left(\frac{Y}{N}\right)+\zeta \left(\frac{S}{N}\right)-\beta (1+\delta_{1}+\delta_{2})\left(\frac{Z}{N}\right)\right]+\lambda_{1}\zeta Z\left(\frac{S}{N}\right)\\ \frac{dY}{dt}=Y\left[-\mu -\alpha +\beta \left(\frac{S}{N}\right)-\zeta \left(\frac{Z+C}{N}\right)\right]+\gamma Z+(\beta (1+\delta_{2})-\lambda_{2}\zeta )Z\left(\frac{S}{N}\right)\\ \frac{dZ}{dt}=Z\left[-\mu -\gamma -\alpha +\beta (1+\delta_{1}+\delta_{2})\left(\frac{C}{N}\right)+\zeta \left(\frac{Y}{N}\right)+\lambda_{2}\zeta \left(\frac{S}{N}\right)\right]+(\beta (1+\delta_{1})+\zeta )C\left(\frac{Y}{N}\right). \end{array}$$

As is expected, when the parameters which do not appear in the two-patch model (Figure 1a) are taken to be zero, the four-patch cofactor system reduces to the two-patch model, taking *I*(*t*) = *Y *(*t*) + *Z*(*t*), the total number of individuals infected by the primary disease. This phenomenon persists throughout the analysis, and is reflective of the strong analogy between these two models.

### Estimation of simulation parameters

#### Parameter choices for the two-patch model

Since we use the two-patch model to test the validity of the cofactor model in the simulations, we chose values for the parameters in the four-patch cofactor model which are inherited from the former to be within the ranges calculated by Anderson et. al. (1988) [[18], and refs within]. The resulting choices for *μ, α, ν, ϵ *and *β*_*T *_are shown in Table 2. Notably our choice for *β*_*T *_(total transmission rate) represents the lower limit of the range estimated in Anderson et. al. (1988) [18]. This choice was made because even this smallest value of *β*_*T *_results in a declining population under their model. As a result, our calculation of the potential impact of the cofactor intervention should be an underestimate of the true impact. Because this estimated range of *β*_*T *_results in declining population under the two-patch model, use of the lower limit does not cause any loss of generality due to qualitative differences in the pre-intervention global behavior of the population (as compared to larger values of *β*_*T *_).

**Table 2:** Parameter values used in simulations of HIV/schistosomiasis disease system.

two-patch model parameter values:	
*μ*	0.02
*α*	0.04
*ν*	0.06
*β_T_*	0.2
*β_D_*	0.08
*ϵ*	0.5
	
four-patch cofactor model parameter values:	

*μ*	0.02
*α*	0.04
*ν*	0.06
*ϵ*	0.5
*β_D_*	0.08
*ζ*	0.2
γ	0.0-0.2
*δ*_1_	3.0
*δ*_2_	2.0
*λ*_1_	1.0
*λ*_2_	0.0

Parameter values used in simulations of HIV/schistosomiasis disease system.

#### Estimating HIV direct transmission rates (*β*_*D*_)

We estimated *β*_*D *_(direct transmission rate) using two complementary methods. Our first method is based on measurements of rates of HIV incidence and prevalence, which we took from the United States population of military personnel (0.14-0.16 per thousand, [21]) and from childbearing women in the United States (1.5-1.7 per thousand, [22]). Because we are specifically targeting a cofactor-free HIV transmission rate, we used data from populations that were likely to be a affected by as few unmeasured cofactors as possible. This method yields estimates for the direct HIV transmission rate in the range of 0.08 to 0.11 per year. In order to estimate the direct HIV transmission rate in the absence of all cofactors as conservatively as possible, we choose *β*_*D *_to be 0.08 per year in the simulations, the bottom of this estimated range.

The second method derives an estimate for the direct HIV transmission rate from (1) data on the risk of transmission per coital act and the frequency of coital acts for each stage of HIV progression within an infected individual, and (2) the new sexual partner acquisition rate among the low-risk population as reported in [7]. The resulting estimate from this method for the direct HIV transmission rate is 0.11 per year. This lends further support to the choice of a direct transmission rate for use in the simulations which is less than half of the total transmission rate estimates taken from Anderson et. al. (1988) [18] of 0.2 to 0.6 per year.

#### Cofactor rates of transmission and recovery (*ζ *and *γ*)

In the four-patch cofactor model, when the primary disease is absent and the cofactor condition exists in stable equilibrium, the cofactor rate of spread less its recovery rate is larger than the overall population growth rate. In this case, the cofactor-bearing proportion of the population stabilizes at (*ζ *- *γ *- *ν*)/*ζ*. In the absence of intervention, we assume that *γ *= 0 during the time period prior to the invasion of the primary disease, which would correspond to the scenario in which individuals either do not recover from schistosomiasis, or the recovery period is on the order of magnitude of years. We assume that the cofactor, *Schistosoma haematobium*, exists at a stable endemic level in many sub-Saharan African populations. Therefore, existing measurements of the prevalence of this condition in such populations may be taken to be equal to the computed stable proportion. Because *ν *has been chosen separately (see above), this results in an equation which may be solved for *ζ *in terms of the measured prevalence data.

**Figure 2:**
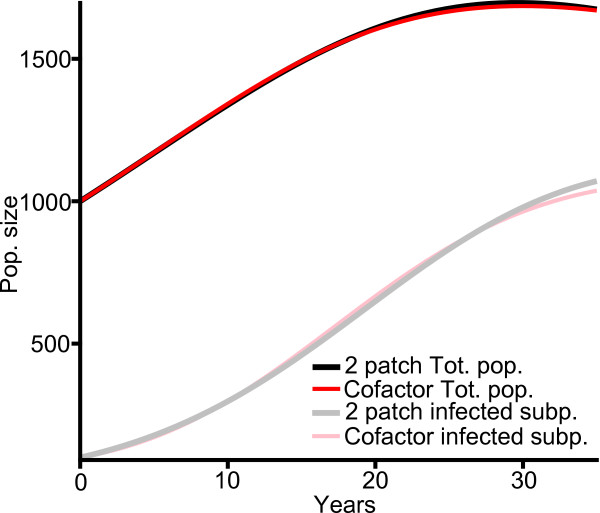
**Agreement of four-patch cofactor model with two-patch model**. 35 year population timeseries (total population, black/red lines; infected subpopulation, gray/pink lines). Two-patch model (black/gray) with *β *= *β*_*T *_; four-patch cofactor model (red/pink) with *β *= *β*_*D*_; other parameters shown in Table 2. Starting conditions: 1000 individuals, 700 carrying the cofactor, 100 infected by the primary disease.

We estimated *ζ *from populations in sub-Saharan Africa from villages in which both incidence and prevalence data were available for the same time period [23,24], focusing on whole populations rather than children only. Since the data on prevalence of schistosomiasis in the literature ranges conservatively from 25% to 75%, any choice of *ζ *which falls between 1.33 and 3 times the size of the population birth rate (*v*) could be considered appropriate for use in our model simulations. The *ζ *value chosen for use in these simulations (*ζ *= 0.12 per year) is exactly 2 times the birth rate (*ν *= 0.06 per year). This value of *ζ *corresponds to an endemic proportion of schistosomiasis in the population of 50%, the center of the range of estimates from the literature, and yields simulation results reflected in Figure 2, which demonstrate that the cofactor model compares well with the observed early HIV prevalence.

In the simulations, the range of values used for the rate of recovery from the cofactor (*γ*) was chosen to highlight the potential impact of this rate. Thus, at the lower end of the chosen range, recovery has little effect on the system, while at the upper end of the chosen range, the recovery rate is already large enough that the system is effectively reduced to a cofactor-free condition. It is interesting to note that values of *γ *that are an order of magnitude smaller than the rate of spread of the cofactor are already sufficient to cause a significant difference in the behavior of the overall system.

#### Estimating the extent to which schistosomiasis increases HIV transmission

In our four-patch cofactor model the parameter *λ*_2 _is used to determine the extent to which the primary disease and the cofactor condition may be simultaneously transmitted to a susceptible individual from an individual with both the primary disease and the cofactor. Because HIV is a sexually transmitted disease and schistosomiasis is transmitted by secondary contact with water sources, it is not possible to transmit both HIV and schistosomiasis in a single interaction. Therefore, *λ*_2 _must be chosen to be zero for these simulations.

The non-negative parameters *δ*_1 _and *δ*_2 _represent the increase in susceptibility and in infectivity induced by the presence of the cofactor. Infection with *S. haematobium *increases susceptibility by increased likelihood of exposure through breached skin at the sites of genital lesions caused by the schistosome. Women with *S. haematobium *are estimated to be three to six times more likely to contract HIV than schistosome-free women [[9], and refs within]. The *S. haematobium *infection also increases HIV titer, resulting in a greater potential infectivity: HIV positive men with genital schistosomiasis have between two and seven fold higher HIV titers in their blood and their semen than schistosome-free men [[9], and refs within]. Susceptibility effects in men and infectivity effects in women may be smaller than those described above, but are likely to also be substantial. In our simulations we therefore estimate *δ*_1 _to be 3.0 and *δ*_2 _to be 2.0, and note that the qualitative aspects of the simulation results were not sensitive to small variations (± 1.0) in these parameters. It is worth noting that the magnitudes of *δ*_1 _and *δ*_2 _estimated here are on the same order as the effect that HSV-2 has on acquisition of HIV [8].

## Results and Discussion

The four-patch cofactor model presented here builds upon and inherits all of the parameter names (*μ, α, β, v, ϵ *Table 1) from the two-patch SI model of Anderson et. al. (1988) [18] describing the impact of HIV on overall host population growth in developing countries. That model tracks the susceptible (*X*(*t*)) and infected (*I*(*t*)) subpopulations of the host population (*N*(*t*)) through time (Figure 1a), and is completely tractable from an analytic viewpoint. Incorporation of the cofactor into this basic model framework requires two new population patches (Figure 1b). The susceptible subpopulation is now divided into *S*(*t*) - susceptible to the primary disease and to the cofactor, and *C*(*t*) - susceptible to the primary disease with the cofactor already present. The infected subpopulation is now divided into *Y*(*t*) - infected by the primary disease but not by the cofactor, and *Z*(*t*) - infected by the primary disease and possessing the cofactor. The expanded model includes parameters controlling differences in the infectivity of and susceptibility to the primary infection caused by the cofactor (non-negative parameters *δ*_2 _and *δ*_1_, respectively, Table 1), as well as parameters for the rates of transmission and loss of the cofactor (*ζ *and *γ*, respectively, Table 1).

Whereas the two-patch model has a single transition (Figure 1a), the four-patch cofactor model includes eight new transitions among subpopulations (Figure 1b). These new transitions arise because the cofactor can be cured, introducing bidirectionality; because the two diseases can be transmitted either singly or together; and because of the greater number of players in the system. Therefore, while the two-patch cofactor-free model admits an exact solution, the additional non-linearity in the four-patch cofactor model makes mathematical analysis of the latter much more difficult. Complete results pertaining to the circumstances under which the primary disease can successfully invade a population in the four-patch cofactor model have been obtained, and are described in the next subsection. We then proceed to apply the four-patch cofactor model through simulations of a specific disease/cofactor system.

### Primary disease invasion in the analytical model

#### Invading the disease-free/cofactor-free state

Suppose that the primary disease agent attempts to invade a population which is entirely free of both the primary disease and the cofactor. In the two-patch model, this invasion occurs through the introduction of one individual who is infected by the primary disease (from population *I*, Figure 1a). In that model, the primary disease successfully invades when the number of new *I*s (susceptible individuals infected) produced by an invading *I *individual (i.e. the basic reproductive number) is greater than one. In the four-patch cofactor model, the primary disease may invade through the introduction of an individual who is infected either by the primary disease alone (from subpopulation *Y *, Figure 1b) or by both the primary disease and the cofactor (subpopulation *Z*, Figure 1b). Analysis of the Jacobian matrix of the linearization of the subsystem for *Y *and *Z *[25] shows that because an invading *Y *cannot produce *Z*s in the absence of *C*s, the primary disease successfully invades when either the number of new *Y*s produced from an invading *Y *or the number of new *Z*s produced from an invading *Z *is greater than one (Appendix, first subsection). This leads to an *R*_0_(0) (the basic reproductive number of the population which starts with no individuals possessing the cofactor) value of max{*β*, λ_2_ζ - γ}/(*μ+α*).

Since patches *Z *and *C *in the four-patch cofactor model are initially empty, for an invasion into a disease-free and cofactor-free population the basic reproductive number of the two-patch model is the same as the number of new *Y*s produced from an invading *Y *in the four-patch cofactor model. Therefore, it is never harder for the primary disease to invade the disease-free/cofactor-free population in the four-patch cofactor model when compared with the two-patch model. In other words, our *R*_0_(0) is at least as large as the basic reproductive number of the two-patch Anderson et. al. (1988) model. The ability of the primary disease to invade the disease-free/cofactor-free population is facilitated by the cofactor when the basic reproductive number of the two-patch model is less than one, and the number of *Z*s produced from an invading *Z *is greater than one. This occurs when (λ_2_*ζ *- γ)/(*μ *+ *α*) > 1. Keeping in mind that *λ*_2_*ζ *is the rate at which *S*s convert to *Z*s, we see that except in the event that the disease and the cofactor are transmitted together substantially more often than singly during a single interaction (or λ_2_*ζ > β*), the cofactor will have no effect on the ability of the infection to invade the disease-free/cofactor-free population.

It is also possible for the primary disease to invade an exponentially growing (*ν *>*μ*) disease-free (*Y *+ *Z *= 0) population even though the proportion of infected individuals approaches zero. By analyzing the Jacobian matrix of the proportions *Y*/*N *and *Z*/*N *of individuals with primary infection only and of individuals with primary infection and the cofactor near the disease and cofactor free state, we find that the proportion of individuals with the primary disease always approaches zero unless *R*_0_(0) > (*ν+α*)/(*μ+α*) >1. This means that the percentage of the population with the primary infection will only become bounded away from zero in the long run when the disease grows fast enough to outpace not only the general death rate, *μ*, but also the population growth rate, *ν*. [18,26]

#### Invading a cofactor endemic population

The primary disease could be introduced to a population in which a cofactor is already established. This would occur if an emerging disease arises in a population in which an established behavior, condition or pathogen exists. For HIV, this scenario best represents the conditions under which the primary disease was introduced into most human populations. In this case it is still true that if either the number of new *Y*s produced from an invading *Y *or the number of new *Z*s produced from an invading *Z *is greater than one, then the primary disease will successfully invade the population (Appendix, second subsection). Since, in the presence of *C*s, the *Z*s can produce *Y*s and the *Y*s can produce *Z*s, the off-diagonal terms in the Jacobian matrix of the linearization of the *Y*-*Z *subsystem are now both non-zero. This produces a scenario in which invasion may occur even when both of these numbers (*Y*s from a *Y *and *Z*s from a *Z*) are smaller than one. The condition governing this type of invasion is complex and not easily interpreted in biological terms (Appendix, second subsection).

Despite this complication, we analytically verified (Appendix, third subsection) that whenever *R*_0_(0) > 1, then the basic reproductive number of the population which starts out with the stable proportion c* of individuals possessing the cofactor, *R*_0_(c*), is also greater than one, meaning that the disease-free/cofactor-*endemic *population can also be invaded by the primary disease. We show that because *R*_0_(c*) ≥ *R*_0_(0), it is never the case that the disease-free/cofactor-free population can be invaded, while the disease-free/cofactor-endemic population cannot. This supports the idea that the cofactor cannot exhibit a preventative effect on disease invasion in this model. The demonstration of this inequality is somewhat subtle, since the starting equilibrium state affects the number of new *Y*s that can be produced by an invading *Y*. During an invasion of the disease-free/cofactor-endemic population, this number is smaller than in an invasion of the disease-free/cofactor-free population due to the smaller available proportion of cofactor-free susceptibles (*S*s) in the population.

When the impact of the cofactor (as measured by *δ*_1 _and *δ*_2_) is small, whenever the disease-free/cofactor-free population resists invasion the disease-free/cofactor-endemic population will also resist invasion by the primary infection. This means that when the basic reproductive number of the two-patch model is smaller than one, there will be a domain of values of *δ*_1 _and *δ*_2 _for which *R*_0_(c*) < 1 as well. However, if the impact of the cofactor is sufficiently large, *R*_0_(c*) will be larger than one and the primary disease will successfully invade the disease-free/cofactor-endemic population even when it would fail to invade the disease-free/cofactor-free population. Specifically, infection of cofactor-possessing individuals (C to Z transitions) can be quite common in the disease-free/cofactor-endemic case. This group has the potential to have the largest transmission rate in the system, because both the risk of infection in the *C *individual and the infectivity of the *Z *individual have been increased by the cofactor condition. As a result each invading *Z *may produce more new *Z*s than an invading *Y *can produce *Y*s, and will in fact produce more than one new *Z *when the values of *δ*_1 _and/or *δ*_2 _are sufficiently large. The precise criteria for this scenario appears in the second subsection of the Appendix.

In typical ecological models, having once come to understand the boundary equilibria (in which one subpopulation is absent), the analysis would proceed via persistence theory [27,28] to consider the existence of a coexistence equilibrium state at which all subpopulations are present. However, the four-patch cofactor model is somewhat unusual among ecological models because it includes so many intrinsic interactions among subpopulations. As a result persistence theory cannot be applied, and it is impossible to explicitly solve the general system or to precisely determine the long term behavior of the solutions. It is even difficult to locate (in terms of the parameters) an equilibrium concentration state in which the cofactor-only (*C*s), the primary disease-only (*Y*s), and the cofactor with primary disease subpopulations (*Z*s), are all present. This situation likely results from the interactive nature of the *Y *and *Z *populations - that is, *Z*s create *Y*s from interacting with *S*s and *Y*s create *Z*s from interacting with *C*s. It is the non-linear terms in the model which result from these interactions that prevent the model from being solved in closed form for the positive equilibrium state. (Note that the model does admit a positive equilibrium, but that it cannot be found analytically.)

Timeseries simulations of the model over a variety of parameter ranges have provided no hint that the model can exhibit either oscillatory or chaotic behavior in its component subpopulations. It seems that whenever the primary disease can successfully invade the population in the four-patch cofactor model, then the proportions of each of the subpopulations will tend toward a positive equilibrium value. This means that even though it is not possible to precisely predict the long term behavior of the system in general terms, the model will still have great predictive value through simulations with a given set of parameter values.

The comparison of the four-patch cofactor model with the two-patch model highlights the important analytical changes that occur in this system as a result of the addition of a single player: a linear and explicitly solvable system becomes non-linear and unsolvable. This qualitative change in the system as a result of the presence of a cofactor indicates that in populations where a potential cofactor co-occurs with a primary disease of interest, management approaches based on parallel and independent models can be expected to considerably misrepresent potential effects of interventions to the spread of primary infection. In particular, in populations that are completely naive to both the primary disease and the cofactor and in populations in which a cofactor is established at the time of invasion by a primary disease, a simple, cofactor-free model like that of Anderson et. al. [18] can substantially overestimate the invasion force of the primary infection by attributing the entire rate of spread of the infection to the infection itself, rather than to the combined force of the primary infection and its assisting cofactor.

### Simulation of HIV with *Schistosoma haematobium *cofactor

We further investigate the effect of cofactors on the spread and persistence of a primary disease and the potential impact of intervention policies targeting cofactors rather than the primary disease through simulations. In these simulations, estimates of transmission contact rates for the primary disease (*β*) and the cofactor (*ζ*) are based on those of HIV and *Schistosoma haematobium*, the causal agent of genital schistosomiasis. We chose the cofactor *S*. *haematobium *for this study because it is endemic in many areas with high HIV prevalence [9,29], has been identified as a cofactor for HIV [30-32], and is easily and inexpensively treatable [9,33].

In Anderson et. al. (1988) [[18], and refs within], many demographic parameters are chosen directly from measurement data of birth and death rates in the sub-Saharan region which comprises the focus of that study. They also estimated what we call the total transmission rate at the start of the HIV epidemic (*β*_*T *_= *β*, Figure 1a) in such a way that the output from their model would match HIV prevalence data from those same locales. By contrast, the four-patch cofactor model makes use of a direct transmission rate (*β*_D _= *β*, Figure 1b) from which any effect of the cofactor has been removed. For the following simulations we estimated *β*_D_, *β*_*T *_, and other HIV-specific parameters using epidemiological data and estimates from HIV-specific models [[7,18,21,22] details in Methods]. Since the schistosomiasis incidence and prevalence data [23,24] provide a broad range of estimates for schistosome transmission rates (*ζ*), we choose *ζ *in the center of the estimated range with the result that the timeseries of the total number of individuals infected by the primary disease under the four-patch cofactor model (with *β*_D_) agrees well with the total number of individuals infected by the primary disease under the two-patch model (with *β*_*T *_). All of these parameters are fixed for the following simulations (Table 2, values chosen; methods section, motivation for choices).

The quantitative results of the following simulations are based on very basic models of both HIV and schistosomiasis which suppress many significant details of both conditions. Consequently, the focus here is on the qualitative results of the simulations under the parameter choices that have been made, illustrating the possible importance of the disease/cofactor synergy on the primary disease progression within a population. In particular, the primary importance of the two-patch model of Anderson et. al. (1988) is its ability to give an early indication, based on sparse observational data during the early stages of an epidemic, of the potential demographic impact of the epidemic. With the goal of qualitative evaluation of cofactor impact during the same early stages of such an epidemic in mind, it is therefore reasonable to base both the construction of the four-patch cofactor model and the selection of parameter values for use in simulation on the structure and parameter choices made in that two-patch model.

#### Agreement of the four-patch cofactor model with two-patch model results

If the four-patch cofactor model is to be used to measure the decrease in HIV prevalence resulting from a treatment program for a cofactor condition, it should first be verified that it models the total population and the actual number of infected individuals under the conditions where the cofactor is present in the population and no recovery from the cofactor takes place (*γ *= 0) in a way which is comparable to the two-patch model. For this comparison, in the four-patch cofactor model simulation we allow individuals to occupy all four patches (*S*, *C*, *Y *and *Z*), set *β*_*D *_= 0.08 per year, and the other parameters as listed in Table 2. In contrast, in the two-patch solution, *β*_*T *_is set to 0.2 per year, with the other two-patch parameters the same as the analogous four-patch parameter values. The parameter *β*_*T *_is always at least as large as *β*_*D *_because it implicitly incorporates transitions involving *Z*s as well as those involving *Y*s. Figure 2 shows the timeseries of the total population and the number of infected individuals for both the four-patch model (in red), and the two-patch model (in black). The similarity of these timeseries indicates that the four-patch model does indeed correctly model the total population and the actual number of infected individuals under our selection of parameters as compared to the two-patch model.

#### The HIV prevalence in a cofactor-free population

In order to determine whether or not the four-patch cofactor model correctly measures the decrease in HIV prevalence resulting from a treatment program for a cofactor condition, it is necessary to estimate the expected HIV prevalence in a cofactor-free population under the two-patch model. In a truly cofactor-free population the total transmission rate *β*_*T *_would simply be equal to the direct transmission rate *β*_*D*_. Therefore, to measure the difference in prevalence of HIV between the observed cofactor-endemic and a truly cofactor-free context, we compare the solutions of the two-patch model using the total transmission rate (with *β *= *β*_*T *_= 0.2 as the HIV transmission rate) just as above, and then using our estimated direct transmission rate (*β *= *β*_D _= 0.08).

**Figure 3:**
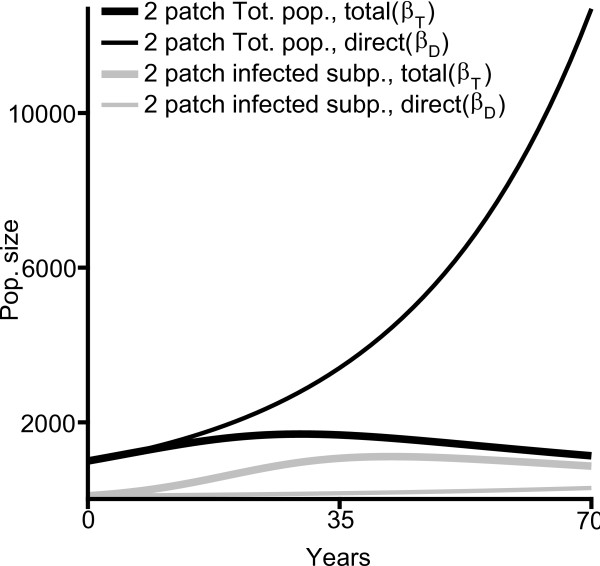
**HIV prevalence in a cofactor free population**. 35 year population time series for two-patch model with total transmission rate (*β*_*T *_, thick lines) and two-patch model with direct transmission rate (*β*_*D*_, thin lines). Total populations indicated in black, infected subpopulations in gray. Starting conditions: 1000 individuals, 100 infected by the primary disease, 0 carrying the cofactor.

**Figure 4:**
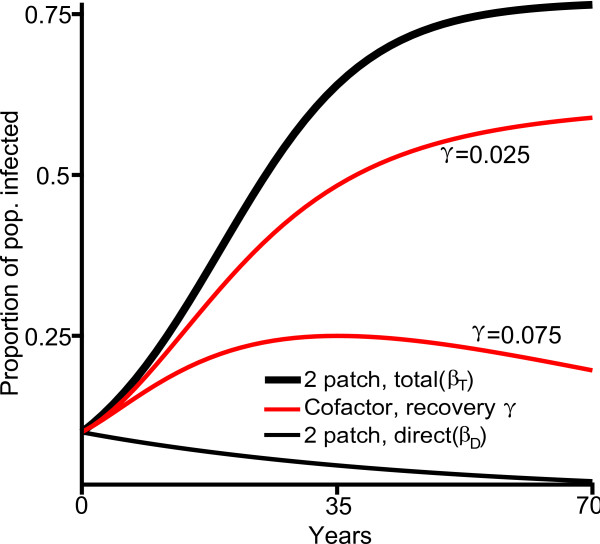
**The effect of cofactor intervention on HIV prevalence**. The timeseries for the concentration of infected individuals within the population under the two-patch model with both total (thick black line) and direct transmission rates (thin black line) and under the cofactor model with direct transmission rate (*β_D_*) and cofactor recovery rates *γ *= 0.025 and *γ *= 0.075 (both in red). This timeseries covers a 70 year period, starting with 1000 individuals, of whom 100 are infected by the primary disease (subpopulation Y, Figure 1b) at time zero and 700 carry the cofactor condition (subpopulation C, Figure 1b) at time zero.

Figure 3 shows timeseries for the total population and the number of infected individuals for two two-patch model solutions. The total transmission curve uses the estimate of the total transmission rate, *β*_*T *_(used in the above model), as the estimate of the parameter *β*. The direct transmission curve uses the estimated direct transmission rate, *β*_*D*_, as the estimate of *β*. Under our choices of these parameters, the relative proportion of infected individuals is approximately stable (or near the positive equilibrium) after simulating a time period of 70 years. The contrast between the curves in Figure 3 indicates that cofactor conditions can greatly exacerbate the negative impact of the HIV epidemic on a population. The simulated proportion of the population with HIV is near 75% by 70 years under the total transmission rate while under the direct transmission rate the number infected increases at a much slower rate, and the concentration of the infection decreases to near zero by 70 years (Figure 4, black). In addition, while the total transmission rate results in population decline after only 25 years the direct transmission rate allows the population to continue to grow. A program for treating cofactor conditions within a population where the true direct transmission rate is lower than the total transmission rate, as modeled here, might therefore offer the significant benefit of reducing HIV prevalence.

#### The effect of cofactor intervention on HIV prevalence

We now investigate the predicted decrease in HIV prevalence resulting from a treatment program for a cofactor condition in the four-patch cofactor model. We expect to see four-patch timeseries results which fall between the two different two-patch curves in Figure 4 (thick and thin black), with the exact location depending on the magnitude of the treatement effort (as measured by the induced cofactor recovery rate *γ*). To test this, we simulate the four-patch cofactor model using exactly the same collection of parameters as above (Table 2, Figure 2) but now include a positive rate of recovery from the cofactor (*γ *> 0). The total population timeseries from two such simulations (*γ *= 0.025 and *γ *= 0.075) appear in Figure 4 (red), along with the two-patch direct and total transmission rate curves. The simulation with a greater intervention parameter (*γ *= 0.075) shows spread of primary disease more like the two-patch direct transmission rate curve while the simulation with the smaller intervention parameter (*γ *= 0.025) is closer to the two-patch total transmission rate curve. Therefore, the four-patch model does serve to interpolate between the different two-patch curves in a way which depends on the force of the intervention.

**Figure 5:**
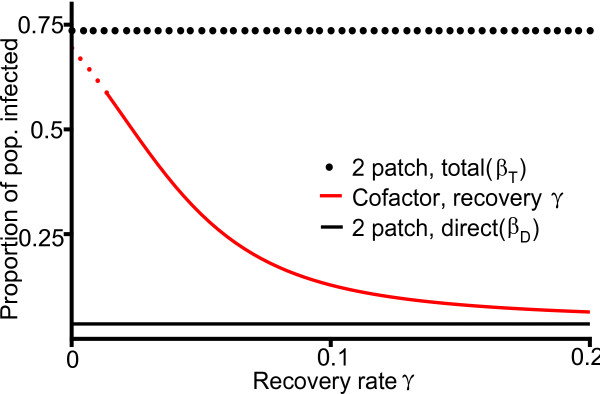
**The impact of the cofactor recovery rate on HIV prevalence**. A 25 year snapshot of the concentration of infected individuals in the population under the two-patch model with both total and direct transmission rates (black horizontal lines above and below) and under the four-patch cofactor model (in red) with direct transmission and cofactor recovery at rate γ. Dotted lines indicate total population is in decline, solid lines indicate population is growing. Plot constructed from 60 simulations initiated with 1000 individuals, of whom 100 are infected by the primary disease (subpopulation Y, Figure 1b) at time zero and 700 carry the cofactor condition (subpopulation C, Figure 1b) at time zero, over which γ, cofactor recovery rate, was varied.

We also simulate the four-patch cofactor model many times over a range of positive rates of recovery from the cofactor (*γ *from 0 to 0.2) to explore the effects of intervention over a range of intensities (Figure 5), focusing on the final concentration of infected individuals after 25 years from each of the simulations and comparing it to the results from the two-patch model with both total and direct transmission rates. Under the total rate in the two-patch model and under low rates of intervention in the four-patch model the total populations are in decline (dotted portions of the curves, Figure 5). Higher rates of intervention, like the direct transmission rate from the two-patch model, result in continued population growth (solid portions of the curves, Figure 5). Notably, when the recovery rate from the cofactor is near zero, the infection concentration from the four-patch cofactor model is quite similar to the infection concentration from the two-patch model with total transmission rates, and as the recovery rate increases, the infection concentration moves closer to that observed under the direct transmission rates, just as suggested by the simulation from Figure 4. Figure 5 can be used to directly estimate the difference in the expected HIV prevalence between a two-patch model system and a four-patch system including treatment of the cofactor at a given constant rate during a 25 year time period.

## Conclusions

Most disease models are developed for the purposes of predicting the impact that the disease will have on the population and providing information relevant to efforts to prevent or reduce that impact. This has lead to a progression in which the disease dynamics are first modelled in a way that treats each individual in the population as identically susceptible to infection by the disease and identically capable of transmitting that infection to other susceptible individuals. As more information becomes available or as the need for intervention grows, more complex stratified models are constructed which take into account differences in risk of infection and transmission by individuals in the population. However, these models do not typically consider those individuals with greater risk of infection or transmission as possessing some further characteristic which itself might be spreading throughout the population, e.g. a cofactor.

It is precisely this consideration, the presence of an increased risk of infection and transmission which itself spreads through the population, which sets the model presented here apart. Other efforts that have been made to understand cofactor-disease scenarios have been aimed at making specific predictions, requiring them to be intensely complex analytically (e.g. [7,8]). These models have had some success in demonstrating that cofactors are important to their particular scenarios, but, without the mathematical analyses that their complexity prohibits, they can lend only vague support to the theory that cofactors may play an important role in many disease systems. In contrast, our simple, general model allows direct evaluation of the the dynamics which result from considering the cofactor to be a spreading condition. Our approach is similar to the generational model analyzed by Diekmann et. al. (1990) [34] which provided a more general method for modelling heterogeneity among susceptibles for the context of diseases with discrete generations.

There are several ways in which the model presented here might be extended to treat more types of disease/cofactor pairs. Modeling the cofactor as a condition which spreads as a direct rate, instead of a density-dependant contact rate, would allow the model to take into account cofactors such as malnutrition or genetic variability, for example. There are also two other extensions which would shift the focus of the model away from our focus on the dynamics of the primary disease only and toward understanding how the primary disease affects the dynamics of the cofactor. First, the model could be extended to consider how the rate of spread of the cofactor depends on the primary disease. Second, the model could allow the introduction of an additional cofactor related death rate. These extensions would more accurately model cofactor dynamics of parasitic conditions like schistosomiasis when interacting with the primary disease HIV, as has been done for the parasites alone in Lloyd-Smith et. al. (2008) [35], and for malaria and tuberculosis using stratified models [16,17].

Because the positive equilibrium proportions of the subpopulations in this model cannot be given explicitly in terms of the parameters, simulations are required to measure the effects of changes to the parameter values on the behavior of the system. To guide the choices of parameter values for use in the simulations, we have chosen an example disease/cofactor pair. The results of these simulations on the HIV/*S*. *haematobium *pair suggests a novel course of action for HIV prevention in the context of host populations like those where genital schistosomiasis is common, i.e. where the cofactor is widespread and *β*_*T *_is substantially greater than *β*_*D*_. This result demonstrates that consideration of cofactors through a simple general model like this one could be an important first step in determining the modeling direction (primary-only vs. complex cofactor) for models to be used in the development of management strategies.

## Appendix - Analysis of Analytical Model

Below we describe the mathematical analysis supporting the theoretical results reported above (Results and Discussion). We begin with the stability analysis of the disease-free/cofactor-free population and continue with the analogous analysis of the disease-free/cofactor-endemic population. The demonstration that instability in the disease-free/cofactor-free population always implies instability in the disease-free/cofactor-endemic population requires the most careful analysis, and it forms the last portion of the section.

### Invasion of a disease-free/cofactor-free population

The *C*-*Y*-*Z *subsystem of the full system (1) has a trivial equilibrium at state *E*_0 _= (0, 0, 0) in which the population is entirely susceptible to the primary infection and no individuals possess the cofactor. Using linear stability analysis, we obtained conditions for successful invasion of the primary disease to *E*_0_. We found that when the individuals infected with the primary disease (*Y*) or with both the primary and cofactor (*Z*) invade *E*_0_, the criterion for the initial establishment of the primary disease is given by

$$\max\{\beta ,\lambda_{2}\zeta -\gamma \}>\mu +\alpha .$$

Since there are no individuals possessing the cofactor in the system initially, if the system is invaded by an individual with the primary infection only (from *Y*), no new individuals with both the primary infection and the cofactor may be produced (into *Z*), resulting in a triangular Jacobian matrix of the linearization of the *Y*-*Z *subsystem near the equilibrium,

$$\left(\begin{array}{cc} \beta -\mu -\alpha & \beta \left(1+\delta_{2}\right)-(\lambda_{2}\zeta -\gamma )\\ 0 & \lambda_{2}\zeta -\gamma -\mu -\alpha \end{array}\right),$$

whose largest eigenvalue is simply the maximum of the diagonal elements.

This condition also arises naturally from considering that the expression *β*/(*μ *+ *α*) represents the number of individuals with the primary infection only (*Y*) that are created from one individual in *Y *within a totally susceptible population, and (λ_2_*ζ *- γ)/(*μ *+ *α*) is the number of individuals with both the cofactor and the primary infection (in *Z*) that are created by one *Z *individual within a totally susceptible population. Thus, if either of these is greater than one, the total number of individuals with either the primary infection only, or the primary infection and the cofactor, must persist within the population. This means that *R*_0_(0), the basic reproductive number for the cofactor system at *E*_0 _is

(2) $$R_{0}(0)=\frac{\max\{\beta ,\lambda_{2}\zeta -\gamma \}}{\mu +\alpha }.$$

It is therefore easier for the primary disease to invade a population under the four-patch cofactor model than under the two patch model when λ_2_*ζ *- γ >*β*. It is never harder for the primary disease to invade in the four-patch model than in the two-patch model.

It is also possible to consider the system of concentration equations for *c *= *C*/*N*, *y *= *Y*/*N*, and *z *= *Z*/*N*, with *s *= *S*/*N*,

(3) $$\begin{array}{l} \frac{dc}{dt}=c\left[-\nu -\gamma +\tau (y+z)-\beta (1+\delta_{1})y+\zeta s-\beta (1+\delta_{1}+\delta_{2})z\right]+\lambda_{1}\zeta zs\\ \frac{dy}{dt}=y\left[-\nu -\alpha +\tau (y+z)+\beta s-\zeta (z+c)\right]+\gamma z+(\beta (1+\delta_{2})-\lambda_{2}\zeta )zs\\ \frac{dz}{dt}=z\left[-\nu -\gamma -\alpha +\beta (1+\delta_{1}+\delta_{2})c+\zeta y+\lambda_{2}\zeta s\right]+(\beta (1+\delta_{1})+\zeta )cy. \end{array}$$

By similar linear analysis of the *y-z *subsystem near *c *= 0, *y *= 0, *z *= 0, we obtain a criterion for the initial establishment of a persisting and endemic subpopulation bearing the primary disease within the population (*Y *and/or *Z*) which is given by

$$\max\{\beta ,\lambda_{2}\zeta -\gamma \}>\nu +\alpha ,$$

or by

R0(0)>ν+α μ+α>1.

Notably, when the population experiences exponential growth((*ν *>*μ*), then the system admits parameter sets for which a decreasing fraction of the population may be infected even though the total number of infected individuals is increasing, as we see occurring in Figure 3 (red curves). Busenberg and van den Driessche (1990) [26] address a similar problem concerning a (cofactor-free) SIR model through the use of concentrations with good effect, since the concentration problem there is reduced to a system of two differential equations. Although reducing our system to concentrations has reduced the number of differential equations in the system from four to three, the system is still analytically intractable, affording no global analytical result.

We can give a criterion for extinction of the population in terms of the endemic proportion of individuals infected by the primary disease, y* + z*. However, although we can solve numerically for this proportion for a given set of parameters, it is not possible to give a general formula for this proportion in terms of the parameters. By solving the differential equation for *N *to see what condition on *y *+ *z *causes the derivative of *N *to be negative, indicating population decay, we find that when

$$y*+z*>\frac{\nu -\mu }{\nu (1-\epsilon )+\alpha },$$

then the population will tend to extinction.

### Invasion of a disease-free/cofactor-endemic population

In many cases, the primary infection and the cofactor do not invade the population simultaneously - a more typical scenario would find the primary infection invading a population in which the cofactor is already pervasive. When the rate at which the cofactor spreads is greater than the rate at which individuals recover from the cofactor plus the population birth rate (*ζ > γ + ν*), in the absence of the primary disease, the concentration of individuals within the population possessing the cofactor will stabilize at 1 - (*γ + ν)/ζ *a proportion which we will refer to as c* in the following. The equilibrium of the *Y*-*Z *subsystem corresponding to this scenario exists at state *E*_*c *_= (0, 0), in which both *Y *and *Z *are zero.

In order to describe the conditions under which this equilibrium state may be successfully invaded by the primary disease, it is useful to describe two new quantities $R_{0}^{y}$ and $R_{0}^{z}$. As is suggested by the symbol, $R_{0}^{y}$ represents the average number of new *Y *individuals which are created from one *Y *individual, and $R_{0}^{z}$ represents the average number of new *Z *individuals which are created from one *Z *individual. These quantities may be computed via linear stability analysis, resulting in formulas which have straightforward logical meaning. For example, $R_{0}^{y}$ is the ratio of the rate at which susceptible individuals are infected by interacting with an individual with the primary infection only (*Y *) to the rate at which the *Y *individual is removed from the population, either by natural death, disease related death, or by contracting the cofactor and becoming an individual in the *Z *population. There is no contribution to $R_{0}^{y}$ from individuals who possess the cofactor only, since when an individual who already possesses the cofactor becomes infected with the primary disease, that individual moves to the *Z *population of individuals who are infected by the primary disease and possess the cofactor. The formula for $R_{0}^{z}$ may be similarly interpreted. The formulas for $R_{0}^{y}$ and $R_{0}^{z}$ are

$$\begin{array}{l} R_{0}^{y}\;=\;\frac{\beta (1-c*)}{\mu +\alpha +\zeta c*},\\ R_{0}^{z}\;=\;\frac{\beta (1+\delta_{1}+\delta_{2})c*+\lambda_{2}\zeta (1-c*)}{\mu +\alpha +\gamma }. \end{array}$$

The quantities $R_{0}^{y}$ and $R_{0}^{z}$ are understandably important to the stability analysis of the equilibrium *E*_*c*_, and closely related expressions appear along the main diagonal of the Jacobian matrix of the linearization of the system near the state *E_c_*. This matrix is

$$\left(\begin{array}{cc} a_{11} & a_{12}\\ a_{21} & a_{22} \end{array}\right),$$

Where

$$\begin{array}{l} a_{11}=-\alpha -\zeta c*-\mu +\beta (1-c*)\\ a_{12}=\gamma +(\beta (1+\delta_{2})-\lambda_{2}\zeta )(1-c*)\\ a_{21}=(\beta (1+\delta_{1})+\zeta )c*\\ a_{22}=-\gamma -\alpha -\mu +\beta (1+\delta_{1}+\delta_{2})c*+\lambda_{2}\zeta (1-c*). \end{array}$$

In particular, *a*_11 _> 0 exactly when $R_{0}^{y}$ > 1, and *a*_22 _> 0 exactly when$R_{0}^{z}$ > 1. The equilibrium *E*_*c *_is known to be unstable either when the trace (*a*_11 _+ *a*_22_) of this matrix is positive or when the determinant (*a*_11_*a*_22 _- *a*_12_*a*_21_) is negative. Since the conditions of the model indicate that the off-diagonal elements of this matrix are both positive, as soon as either or both of the diagonal quantites is greater than zero (which is equivalent to either or both of $R_{0}^{y}$ or $R_{0}^{z}$ being greater than one), then either the trace is positive or the determinant is negative and *E*_*c *_is unstable - the primary disease would successfully invade the population. If both $R_{0}^{y}$ and $R_{0}^{z}$ are smaller than one, then this equilibrium is stable (precluding invasion) for sufficiently small values of *δ*_1 _and *δ*_2_, but becomes unstable (allowing invasion) for sufficiently large values of *δ*_1 _and *δ*_2_. However, before the values of *δ*_1 _and *δ*_2 _grow large enough to make the equilibrium unstable by forcing *a*_22 _> 0 (or $R_{0}^{z}$ > 1), they will first force the value of *a*_22 _to become close enough to zero so that *a*_11_*a*_22 _<*a*_12_*a*_21 _holds, demonstrating that there is a parameter regime in which the off-diagonal terms of the Jacobian matrix (resulting from *Z*s producing *Y*s and vice versa) cause invasion even though the on-diagonal terms cannot do so. The precise conditions which describe the transition between stability and instability of *E*_*c *_in terms of the sizes of *δ*_1 _and *δ*_2 _in this case are those which cause the determinant of the Jacobian to be negative by making the product of *a*_11_*a*_22 _<*a*_12_*a*_21_. Note that this condition results in a two-dimensional region of *δ*_1 _and *δ*_2 _values, and the reader may obtain this condition by computing *a*_11_*a*_22 _and *a*_12_*a*_21 _using the definitions of these terms as they appear above.

### Instability of the disease-free/cofactor-free population implies instability of the disease-free/cofactor-endemic population

Since the cofactor may only contribute in a positive way to the rate of spread of the primary disease in this model when *δ*_1 _and *δ*_2 _are non-negative, it seems intuitively clear that whenever the disease-free/cofactor-free equilibrium is invadable by the primary disease, then the disease-free/cofactor-endemic equilibrium should also be invadable. It also seems reasonable to expect that a sufficiently assistive cofactor will cause a primary disease which would not successfully invade a population in the absence of the cofactor to become endemic.

To demonstrate these intuitive claims, suppose first that *E*_0 _is stable, so that the primary disease is not capable of invading the cofactor free population. In this case it may be observed that for small enough *δ*_1 _and *δ*_2_, the determinant of the *E*_*c *_coefficient matrix may be taken to be arbitrarily close to the quantity

(4) $$(\beta -\alpha -\mu )({\text{λ}}_{2}\zeta -\text{γ}-\alpha -\mu \text{)}\text{.}$$

The conditions for the stability of *E*_0 _require that both terms of this product are negative (eqn. (2)), so that the determinant of the *E*_*c *_matrix is positive for small enough *δ*_1 _and *δ*_2_. In fact, this determinant decreases at least linearly in *δ*_1 _and *δ*_2 _when *E*_0 _is stable, so that for large enough *δ*_1 _and *δ*_2_, the determinant becomes negative and *E*_*c *_becomes unstable. This tells us that any primary disease which is not capable of invading a cofactor-free population may be catalyzed into an endemic disease by a sufficiently assistive cofactor under this model.

Next, suppose that *E*_0 _is unstable. This can happen either when *β - α - μ *> 0(equivalently *β*/(*μ+α*) > 1, the rate at which *Y *s replace themselves is greater than one) or when λ_2_ζ - γ *α - μ *> 0 (equivalently (λ_2_ζ - γ)/*α + μ *> 1, the rate at which *Z*s replace themselves is greater than one). If the second condition fails to hold, it follows that $R_{0}^{z}$ must be greater than one in order for c* to be greater than zero. This means that when the second condition fails, either *E*_*c *_is unstable, or *E*_c _does not exist at all.

If, on the other hand, *E*_0 _is unstable because the first condition fails while the second condition holds, a bit more analysis is required to verify that *E*_*c *_is also unstable. *E*_*c *_is, of course, unstable when either of $R_{0}^{y}$ or $R_{0}^{z}$ is greater than one, so we must only consider the case in which both of these is smaller than one. It follows that, when $R_{0}^{y}$ and $R_{0}^{z}$ are both less than one, both diagonal entries in the *E*_*c *_coefficient matrix are negative, and the determinant of this matrix could theoretically be positive. However, we compute that the maximum value with respect to c* of the terms of this determinant which are constant with respect to *δ*_1 _and *δ*_2 _is achieved when c* = 0, and also has the form of (4). But since the first condition fails and the second condition holds, the value of this expression is negative. It may be further checked that whenever $R_{0}^{y}$ is less than one, then all of the terms of the determinant of *E*_*c*_which involve either *δ*_1 _or *δ*_2 _are negative. Thus the entire determinant is negative for all possible c*, and *E*_*c*_must be unstable whenever *E*_0 _is unstable, just as in each of the other cases.

## Authors' contributions

LG designed the four-patch cofactor model, contributed to its mathematical analysis, conducted the simulations, coordinated the research and writing, and participated in manuscript preparation. BL directed and contributed to the the mathematical analysis of the four-patch model and the simulations, and provided general advice and revisions to the manuscript. SR conceived of the project, provided the biological motivation and application of the model, and participated in manuscript preparation. All authors read and approved the final manuscript.

## Pre-publication history

The pre-publication history for this paper can be accessed here:

http://www.biomedcentral.com/1471-2334/10/248/prepub
